# An Underused Competence: Clinical Pharmacists’ Experiences of Writing Referrals in Transitions of Care

**DOI:** 10.3390/pharmacy14050109

**Published:** 2026-07-23

**Authors:** Katarina Wickman, Sara Modig, Cecilia Lenander, Gabriella Caleres

**Affiliations:** 1 Center for Primary Health Care Research, Department of Clinical Sciences Malmö, Lund University, 202 13 Malmö, Sweden; sara.modig@med.lu.se (S.M.); cecilia.lenander@med.lu.se (C.L.); gabriella.caleres@med.lu.se (G.C.); 2 University Clinic Primary Care Skåne, 223 81 Lund, Sweden; 3 Department of Medicines Management and Informatics in Skåne County, 291 89 Kristianstad, Sweden

**Keywords:** clinical pharmacists, medication-related problem, patient safety, referral, hospital, primary health care, qualitative study, transition of care

## Abstract

Older people are at increased risk of medication-related problems, which may be identified by clinical pharmacists during hospital medication reviews. Some problems are better managed in primary care, where follow-up is possible. Medication-related communication by pharmacists may improve information transfer in care transitions, but this practice remains uncommon in Sweden. This qualitative study explored clinical pharmacists’ experiences and perceptions of writing referrals to primary care. Data were collected through four focus groups and three supplementary interviews with 19 clinical pharmacists from hospitals and primary care, and analysed using qualitative content analysis. Eleven participants had experience writing referrals, although none did so regularly. Four categories emerged: *role of the pharmacist*, *current work procedures*, *pharmacist–physician relationship* and *facilitating factors*. Pharmacists believe they can contribute to patient safety through involvement in transitions of care. An overall concept of *an underused competence* permeated the categories. Referrals identifying medication-related problems are hindered by limited pharmacist integration into the healthcare team and limited continuity. Writing referrals was considered a new procedure requiring collaboration with unfamiliar partners. Organisational changes, including increased pharmacist continuity and clearer pharmacist roles, are needed to implement referral practices. Further research is needed to evaluate effects on medication treatment and patient outcomes.

## 1. Introduction

Older people with multimorbidity and polypharmacy are at greater risk of medication-related problems (MRPs), such as medication interactions, poor adherence and adverse effects [1]. To address these challenges, pharmacists are increasingly integrated into healthcare systems in several countries, including the United Kingdom (UK), the United States (US), Australia, the Netherlands and Sweden [2,3,4,5,6,7,8]. Medication reviews, defined as systematic evaluation and optimisation of a patient’s medication treatment, are a well-established method for identifying MRPs [9,10]. In Skåne county, Sweden, clinical pharmacists at hospitals conduct medication reviews for older patients with many medications. These reviews include medication reconciliation, identification of MRPs and recommendations to physicians. In most cases, there is no further clinical pharmacist intervention in ongoing patient care.

Some MRPs are more appropriately managed in primary care, where follow-up can be ensured and an established relationship with a general practitioner (GP) exists, rather than during the short hospital stay. Examples suggest dose tapering or dose escalation that requires long-term monitoring, or situations when the patient has recovered from an unstable condition, facilitating appropriate medicines regimes and goals. Furthermore, pharmacists also need to communicate recommendations aimed at preventing MRPs from arising, as well as follow-up of laboratory results related to medication regimens, among others. However, information transfer regarding medications is a well-known patient safety risk in care transitions due to, for example, unintended medication discrepancies or inadequate information transfer [11,12,13].

Transition of care refers to when a patient moves between healthcare settings, such as hospital admission or discharge. Transition of care also includes movement between various wards within a hospital but is not explored in this study. In countries such as the US and UK, pharmacists are routinely involved in transition of care, whereas this is still not routine in Sweden [3,14]. Interventions may include medication reconciliation at discharge, referrals to community pharmacists for enhanced post-discharge services involving medication reconciliation, discussions of medication changes, and support with medication management. Evidence suggests that pharmacist interventions in care transitions may reduce hospital readmissions. However, studies show inconsistent results [14,15,16]. In parallel, pharmacists are assuming expanded roles in primary care in several countries [2,4,5,17]. For example, Australia has made progress in integrating pharmacists into primary care, where their responsibilities include providing support in care transitions and post-discharge follow-up [4,18]. In Sweden, the number of clinical pharmacists is still limited, and maximising the benefits of available services is important. Given the differences in healthcare systems and conditions, research is needed on what interventions to adopt to improve patient care. Referrals have been suggested as one approach to improve information transfer. Several studies reported improvement in the quality of the discharge process, but inconsistent impact on readmission rates [15,16,19]. Previous studies mainly focus on quality improvement, but less is known about pharmacists’ perspectives.

A referral is the direction of an individual to the appropriate healthcare provider to address relevant healthcare needs [20]. Referral information is directed to the primary care, who carry overall responsibility for ongoing decision-making. Since 2020, clinical pharmacists in southern Sweden have been authorised to write referrals concerning MRPs to GPs attached to the existing discharge documentation and sent by post or fax. These pharmacist referrals concern various information, such as reports from patients regarding adherence problems or adverse effects. They may also concern a request for the GP to assess gradual discontinuation of therapies or diagnoses that need further follow-up and perhaps additional therapy, as well as interactions of clinical significance. Introduction of this work model was delayed due to the pandemic of Coronavirus Disease 2019 (COVID-19) and anecdotal evidence suggests pharmacist referrals have been used to a lesser extent than expected. Swedish studies regarding the pharmacists’ perceptions of the model are lacking. In this qualitative study, we aimed to explore the clinical pharmacists’ experiences and perceptions of writing referrals, as well as the challenges and facilitating factors for this procedure.

## 2. Methods

### 2.1. Study Design

This qualitative study was reported according to the template for Consolidated criteria for Reporting Qualitative research (COREQ) (Supplementary File S1) for qualitative studies [21]. Data were collected through focus group discussions; a suitable method to explore the experiences and views of a group with shared knowledge and experiences. Focus groups typically comprise a small group of participants, ideally few enough to allow all participants to share their views, yet large enough to provide a diversity of perspectives [22]. The qualitative data emerging from the focused discussion are used to understand a specific subject [22]. Supplementary interviews were included both to accommodate participants who were unable to attend the focus group discussions and to enable the formation of balanced focus groups. Although the study topic was not inherently sensitive, care was taken to avoid combining managers and employees within the same group. This approach was intended to promote open dialogue and maximise the likelihood that all perspectives would be voiced. The inclusion of supplementary individual interviews was also valuable in further strengthening the study’s information power.

### 2.2. Setting and Participants

The study was conducted in Skåne county, which at the time had nine hospitals with approximately 2300 inpatient beds (excluding intensive care units), and 160 primary care centres. Of these, eight hospitals and 83 primary care centres were publicly operated. Criterion sampling was applied. The inclusion criterion was employment as a clinical pharmacist in public healthcare. No clinical pharmacists were employed in the private hospital at the time. Clinical pharmacists at the psychiatric hospital (two at the time) were not invited, as this hospital had not introduced the referral process. No other exclusion criteria were applied. All 34 clinical pharmacists employed in public hospitals in November 2022 were invited via email to participate in the study (across nine hospitals), as well as all four clinical pharmacists in public primary healthcare who also had experience with referrals. All clinical pharmacists were participants in a regional network, where their emails were visible. A member of the research team (KW) was also included in the same network. The network worked toward professional development and capacity building, promoting research as well as networking within the group. The clinical pharmacists were invited to participate and were asked to respond to the email if interested. Individuals who did not wish to participate could either actively decline or choose not to respond to the invitation.

A total of 20 clinical pharmacists (henceforth referred to as pharmacists) accepted the invitation, of which one dropped out due to personal reasons. Focus groups were organised geographically (northwest region, middle region, southwest region and northeast region). The composition of the focus groups was primarily guided by geographical considerations and the number of participants (four to six participants per focus group). At the time of recruitment, it was known that there was a distribution of junior and senior staff across geographical areas; however, this was not a determining factor in the recruitment process. An additional benefit of this approach was the inclusion of both junior and senior pharmacists across the groups, except for one. Individual interviews were conducted with participants unable to attend the focus groups as well as to achieve balanced groups.

### 2.3. Data Collection

Three focus group discussions were initially planned. To ensure saturation, a fourth focus group discussion was conducted, but no new data emerged. All focus groups were held face-to-face, but in the fourth focus group one participant attended digitally. A total of 16 pharmacists with varying experience attended the focus group discussions (Table 1). The focus group discussions were conducted from November 2022 to February 2023. Each focus group included four to five participants, except one group with three participants due to late dropout (personal reasons). Nevertheless, the material was perceived to be informative. Three focus group discussions were held in conference rooms at the participants’ workplaces, whereas one took place in a conference room at a university. All managers approved the distribution of the study invitation; however, all practical arrangements, including the venues, were organised independently by the moderator. The employer permitted the focus groups and individual interviews to be conducted on its premises during working hours. The three individual interviews, from February 2023 to April 2023, took place in the respondents’ offices.

A semi-structured interview guide was developed by the authors (Supplementary File S2). The guide aimed to explore the participants’ perceptions and experiences regarding pharmacist referrals. The moderator based the questions on the semi-structured interview guide, but vivid discussions continued among the participants. Given that several pharmacists (eight) lacked experience with referrals, it explicitly included open questions addressing factors that could stimulate engagement in the referral process, as well as potential barriers. One section in the guide focused on pharmacists’ perceptions of their role in patient safety, recognising that transition of care presents a risk situation. A minor structural adjustment was performed after the first focus group discussion to obtain a natural flow in the discussions; the content, however, remained unchanged.

All participants received written information about the study. Information was also given orally, and it was possible to ask questions before the discussions and interviews started. All participants gave written consent. The focus group discussions were moderated by KW and co-moderated by GC, and the individual interviews were conducted by KW. All discussions and interviews were audio recorded and transcribed verbatim. In addition, the co-moderator took notes during the focus group discussions. Due to partial technical difficulties in the recording of one focus group discussion (no. 2), a written summary of the discussion was sent back to the participants for confirmation. No further additions to the summary were needed.

### 2.4. Data Analysis

This qualitative study applied content analysis inspired by Graneheim and Lundman [23,24]. Initially, the focus group discussions and interviews were listened to several times to get a sense of the data and transcribed verbatim. The data were processed with an inductive approach. At first, meaning units were identified, condensed, and then coded by KW. All other authors cross-checked the same coded focus group (no. 2), blinded from each other, to reach consensus and agreement in identifying meaning units and codes. The remaining focus group discussion data was coded by KW and subsequently cross-checked by one research group member per focus group. Thereafter, the whole research group had a discussion based on the impression of the collected data and the codes. The codes were adapted into subcategories. Finally, subcategories were processed, and main categories were established. An overall concept based on the categories emerged. The three interviews were coded and cross-checked in the same manner. This material was analysed focusing on whether anything new emerged. Table 2 demonstrates examples of text condensation and coding.

### 2.5. Pre-Understanding

All authors participated in the analysis and categorisation. KW (moderator) and GC (co-moderator) led the focus group discussions. The moderator, KW, had no previous experience of qualitative research except for a course in the PhD-study programme, but several years of experience as a clinical pharmacist in public hospitals in the county. The co-moderator, the GC, a GP, and a PhD with previous experience in qualitative studies and focus group discussions, took field notes and supported the moderator. The different pre-understandings strengthened the material as the moderator and co-moderator approached with different perspectives. The other research group members, CL (pharmacist and associate professor) and SM (GP and associate professor), had experience from several qualitative studies.

## 3. Results

A total of 11 respondents had experience writing referrals, but none on a regular basis. The participants had experience in various departments such as internal medicine, surgery, orthopaedic, and geriatric clinics. Four main categories emerged from the data: the *role of the pharmacist*, *current work procedures, relationship with physicians* and *facilitating factors* (Table 3). Results from the discussions and interviews were consistent.

### 3.1. Role of the Pharmacist

The respondents’ perspectives in this category concerned the unclear role of pharmacists in healthcare, both regarding new procedures and responsibilities. Taking a new role in interacting with the patients and still not undermining the trust in the physicians, risking adherence problems, was also covered by the participants. The participants also viewed pharmacists’ thoroughness as both a strength and a potential barrier, as it could be perceived as time-consuming. Furthermore, a couple of respondents expressed a desire to move away from traditional stereotypes of pharmacists as primarily serving a control or monitoring function.

The participants described the pharmacist role as unclear to other professions and emphasised the need to clarify pharmacists’ competencies and responsibilities. The distinction between dose-dispensing pharmacists and clinical pharmacists was described as unclear. Although the respondents considered communicating medication-related information part of their role, they lacked authority to modify the medication list in the electronic medical records. The participants stated that writing referrals might strengthen their role and increase autonomy. One participant stated:

*“It’s great for our profession to be able to feel that we have that power, or so to say, to send referrals and that we can do certain things ourselves, in general it’s always the case that we need someone else to do it…”*

(FG2)

Several focus groups expressed that hospital physicians were often unaware that pharmacists could write referrals and therefore rarely requested this service. Participants described writing referrals as a new task that required adaptation to new administrative procedures and forms of professional communication. A lack of experience communicating with external healthcare providers was considered a barrier by some, although participants generally viewed referral writing positively. However, integrating the task into existing workflows was considered challenging. As stated by one respondent:

“*I don’t think anyone is negative about it (referrals), but I guess it’s a new task that… It affects quite a lot in the work we do today… It’s not just to write a referral, but it’s like the whole thing. The entire (work) flow”*

(FG3)

The respondents expressed concerns that discussing certain MRPs might worry patients, negatively affect adherence, or undermine trust in their GP. Some MRPs were considered easier to communicate than others, particularly compared with more sensitive treatment issues. This was exemplified below:

*“Then there are some things that you can’t really say (to the patient), that’s maybe not a suitable (medication treatment)… You (as a pharmacist) must be careful, and you might ruin the adherence if you think out loud”*

(FG3)

The participants also reported that patients’ access to the electronic medical records influenced their documentation practices. Situations where patients’ treatment preferences differed from pharmacists’ recommendations (i.e., inappropriate medicine regimes) to the GP were described as barriers to referrals and were also perceived as time-consuming. Despite these challenges, the participants reported that patients were generally positive towards pharmacist-led medication reviews at the clinic.

The pharmacists described thoroughness as a central aspect of their professional practice and emphasised the importance of providing physicians with complete background information. At the same time, this thoroughness was perceived as time-consuming and could create uncertainty about whether referrals were sufficiently complete to be submitted. As one respondent expressed:

*“That we should dare more. And don’t overthink it, it doesn’t have to be perfect, but…well, send a referral. No, a mediocre referral feels better than nothing”*

(FG2)

Participants further described referrals as a means of improving information transfer and facilitating follow-up of recommendations from hospital-based medication reviews. The pharmacists expressed a desire to contribute through their pharmacotherapeutic expertise rather than being perceived as a control or monitoring function. They described their role as identifying MRPs and providing recommendations while trusting physicians to implement treatment decisions, as illustrated here:

“*I want to contribute with my expertise… I don’t want to be the police or control function; I’m not interested in that”*

(FG2)

### 3.2. Current Work Procedures

Participants in the focus groups and individual interviews described how *current work procedures*, organisational priorities, and managerial support influenced the use of referrals. They reported that priorities varied across departments and were affected by management attitudes, staffing levels and the high prevalence of inaccurate medication lists. Medication reconciliation at admission was described as a well-established and prioritised task, often taking precedence over medication reviews and discharge-related activities. At the same time, participants noted that medication reconciliations sometimes identified MRPs requiring follow-up after discharge. Due to pharmacist shortages, participants described a need to prioritise tasks and expressed a strong desire for increased staffing to expand pharmaceutical services beyond medication reconciliation.

Insufficient pharmacist continuity on hospital wards was expressed as a major barrier by almost all participants. They described how infrequent ward visits limited opportunities to follow up MRPs and assess whether recommendations were relevant to refer to GPs. As several expressed:

*“In a way, you are not fully part of the team, you are welcome, but you’re not involved all the time in the care of the patient, which makes it hard with the timing”*

(FG3)

Several participants emphasised that without continuous involvement, it was difficult to determine whether identified MRPs had already been addressed before discharge.

*“And there’s also the problem that we don’t always monitor the patients during their hospital stay and don’t really have the resources to monitor the patients, so we don’t know if it’s (the MRP) still even relevant when the patient actually goes home”*

(FG4)

*“Our involvement was rather intermittent; we were often present on the ward for only one or two days (weekly). We reviewed newly admitted patients but did not follow them throughout their hospitalisation. Since the issues identified at admission are often managed during the hospital stay, continuity is important. Without being present and able to follow the patient’s progress, it is difficult to determine whether a referral is warranted”*

(FG1).

*“I mainly send them when I’ve had the opportunity to work clinically on a more regular basis. Because if you’re only working clinically one day and then have a lot of meetings, you don’t always have time to remember that you should send a referral. But when you’re working more regularly in a clinical role, it’s much easier to incorporate it into your routine”*

(Individual Interview 1)

Likewise, lack of physician continuity, with inpatients often being managed by different physicians over time, was perceived to hinder collaboration and increase the risk of information loss.

*“On the other hand, in a ward where physicians frequently rotate and a different doctor takes over, you may not know what has already been addressed and what has not. It feels like this requires much more work from the pharmacist to keep track of what has changed and what has not, in order to avoid sending a referral for an issue that has already been resolved”*

(Individual Interview 3)

Pharmacists described actively seeking out physicians to discuss medication-related findings because formal structures for collaboration were often lacking.

Short hospital stays were also seen as a barrier. The participants described difficulties identifying the optimal timing for referrals as they generally preferred sending referrals near discharge to avoid communicating information about already resolved MRPs. However, because pharmacists were often not involved in the discharge process, referrals could be overlooked. Several participants expressed a desire for greater involvement in care transitions. A participant stated:

*“I think it’s really important that we’re involved in all the care transitions really, that we’re involved everywhere and support, because I think we can do a lot of good”*

(FG4)

Many participants expressed concerns that the lack of pharmacist involvement at discharge could result in a plausible risk of misleading discharge information if the (standard) discharge message “medication review conducted during hospital stay” was transferred to primary care, although MRPs remained unsolved without any further request. The absence of a multi-professional discharge routine was also considered a hindering factor regarding pharmacist referrals.

The respondents expressed varying views regarding which MRPs were appropriate for referral. Acute MRPs were generally considered to be managed during hospitalisation, whereas remaining issues and MRPs requiring follow-up after recovery were viewed as more suitable for referral to primary care. Medication management problems, adherence issues, and unclear indications were frequently highlighted as appropriate referral topics. MRPs identified in patients discharged directly from emergency departments were described as particularly important to communicate to primary care.

Some participants also saw value in referrals concerning optimisation of chronic disease treatment, especially for somewhat less frail patients. In contrast, referrals involving complex or multiple MRPs were perceived as more challenging, particularly when recommendations were vague. They also mentioned that patient history may be incomplete in hospitals, which might be a factor to consider in relation to when and where to address MRPs. Well-defined MRPs and clear recommendations were considered to facilitate follow-up and increase the likelihood that recommendations would be acted upon.

Several participants described various alternatives to pharmacist-written referrals. Medication-related recommendations were sometimes communicated through discharge summaries, although participants were uncertain whether these messages were acknowledged by GPs. Another strategy involved asking physicians to write referrals based on identified MRPs or refer to the pharmacist’s documentation. However, participants described uncertainty regarding whether referrals were actually sent. Using the patient as the messenger was also reported.

Despite these alternatives, participants generally viewed referrals as the preferred communication strategy when follow-up action was required, as referrals were perceived as a clearer transfer of responsibility than discharge summaries.

### 3.3. Pharmacist–Physician Relationship

The participants described how the *pharmacist–physician relationship* influenced referral practices. Many described communication with an unfamiliar receiver as a hindering factor. In addition, several pharmacists expressed that the need for referrals was perceived to vary between departments. Physicians in geriatric and internal medicine departments were described as more likely to address MRPs, whereas physicians in surgical departments were perceived to focus primarily on acute issues. Consequently, referrals were considered more important when the pharmacists had less confidence that hospital physicians would address identified MRPs.

Many respondents expressed barriers such as uncertainties regarding the GPs’ situation, such as their previous experience from pharmacist collaboration, concerns about causing an increased workload, and the risk of recommendations being misinterpreted. However, participants also perceived that GPs appreciated pharmacists’ contributions. A referral written with an educational sense—a method used by a few—was believed to stimulate learning and subsequently benefit other patients. However, writing referrals was still seen as a voluntary task, which was avoided by some.

Several pharmacists discussed positive and negative aspects between pharmacist-phrased referrals versus those written by physicians. Many of the respondents preferred writing referrals themselves, as this reduced the risk of recommendations being modified, misunderstood or omitted. One participant expressed:

*“And then, of course, there’s the communication aspect as well. That’s a big advantage for us—we can communicate directly what we want, without having an intermediary in between”*

(Individual Interview 2)

Although physician-written referrals were sometimes viewed as time-saving and potentially advantageous for physician-to-physician communication, participants also described risks of incomplete information or misinterpretation.

*“And in the referral, she (the physician) just wrote [The pharmacist observed there is no indication. Consider discontinuation please.] … And that’s not how I had expressed it myself”*

(FG4)

In settings where pharmacist–physician relationships were less established, physicians were perceived as more likely to write the referrals. Some participants viewed follow-up of MRPs as primarily the physician’s responsibility.

The participants discussed that junior physicians may be more open to accepting recommendations; they were also perceived as less secure in their clinical decision-making and less inclined to act independently, which in turn could influence the referral process. The respondents also expressed concerns that referrals, as written communication only, could be misinterpreted by GPs because they lacked direct dialogue. Furthermore, the respondents stated that established collaboration with hospital physicians was described as facilitating communication and increasing the likelihood that recommendations would be acted upon. One respondent stated that

*“Every time there are new physicians (at the clinic), I try to monitor those first weeks a little more closely, to know that they… that our collaboration functions and that they understand what my role is, and at those times I check if they have written the referral as agreed upon”*

(FG2)

One participant noted that it was sometimes forgotten that primary care and hospitals use separate electronic medical record systems, and that GPs therefore do not routinely review hospital records. As a result, a referral was, therefore, forgotten.

Several focus groups discussed the uncertainty regarding the receiving GP, including whether recommendations would be understood, prioritised or acted upon. Because many pharmacists often were unfamiliar with the recipient, several described referrals as communicating “into a vacuum”. As respondents expressed:

*“I think it’s the aspect of sending it out in a vacuum, you don’t know anything about the receiver, so if I discuss with a physician here at the clinic who I know, then I know how to communicate the information ….in the way I intend to…”*

(FG3)

The participants expressed that limited experience from primary care was considered a barrier to writing referrals. One group described the limited understanding of what happens in primary care as follows:

*“When referring to someone unknown, and you don’t have a sense of how that world works or how the referral will be perceived, writing a referral may become a greater barrier”*

(FG1)

Those who recognised the receiving GPs described feeling more confident about how their recommendations would be perceived and interpreted. They also described difficulties questioning decisions previously made by GPs. Nevertheless, many of the respondents emphasised the importance of communicating identified MRPs for GP assessment. Despite all these uncertainties, the participants generally believed that GPs valued pharmacists’ recommendations and recognised their competence. One pharmacist described a previous situation:

“*When pharmacist statement was introduced in the discharge information (years ago), the GPs were asked about whether this information was of interest and the GPs responded yes, yes, yes”*

(FG3)

And another participant stated:

*“I actually think that primary care would often appreciate it and welcome it when a medication review has been carried out”*

(Individual Interview 3)

The participants expressed uncertainty regarding GPs’ knowledge of hospital pharmacists’ roles, responsibilities and referral practices. They highlighted a need for greater visibility of pharmaceutical services and clearer understanding of roles to facilitate referral practice. One pharmacist expressed:

*“You have no idea if this doctor who receives the referral has collaborated with a pharmacist before”*.

(FG4)

Another commonly described concern was the risk of creating additional workload for the GPs or being perceived as questioning their clinical decisions. The participants also expressed that communicating clear recommendations to GPs could facilitate action instead of vague recommendations. One focus group linked the concerns regarding relationships to previous negative experiences of interprofessional collaboration and to traditional hierarchical relationships between pharmacists and physicians. To avoid conflict, several groups suggested phrasing recommendations as questions rather than directives. Greater awareness of GPs’ generally positive attitudes towards pharmacist input was viewed as a factor that could encourage increased use of referrals.

### 3.4. Facilitating Factors

The participants described several current and future facilitators necessary to support pharmacist referrals, such as clear instructions and introduction. Being integrated into the daily healthcare team was perceived to strengthen understanding of pharmacists’ roles and responsibilities, thereby facilitating referral practices. Participants also emphasised feedback from GPs as an important factor to stimulate pharmacist referrals.

While current instructions for the referral practice were generally perceived as adequate, participants from several interviews highlighted a need for improved routines and introductions, particularly for new employees. As one respondent expressed:

*“I thought it was pretty easy to follow the instructions, I thought it was very clear, and it didn’t take nearly as long as I had feared, so I thought it was smooth, that is my opinion”*

(FG4)

One participant also suggested practical technical improvements, such as the possibility of sending referrals electronically. Support from managers and colleagues, as well as clear goals for referral activities, were described as factors that could encourage the adoption and continuation of the practice.

Being integrated into the daily healthcare team was described as an important facilitator for writing. Participants reported that closer involvement in ongoing patient care could improve communication, clarify pharmacists’ roles and responsibilities, and facilitate collaboration with physicians, nurses and administrative staff. As noted here:

*“It’s always the thing about timing, when to highlight something, what actions to take, since you’re partly outside of the team, you are welcome in the team, but you’re not fully taking part of the care of the patient, so it’s very hard to find the right timing”*

(FG3)

Feedback was stated as a key facilitator for referral writing. Participants described how responses from GPs and encouragement from physicians increased motivation to continue referral practice. The perception that referrals contributed to patient benefit was also described as an important motivating factor. As exemplified here:

*“When I had sent my first referral and told the physician at the ward, he expressed it was great, so the next time when I asked him to adjust something about a bisphosphonate, he asked me to write a referral instead about that too”*

(FG1)

Some participants also suggested that referrals might be easier to write when the recipient was another pharmacist, as intraprofessional communication was perceived as carrying a lower risk of misunderstanding.

## 4. Discussion

This study explored pharmacists’ experiences and perceptions of the referral process.

**Figure 1:**
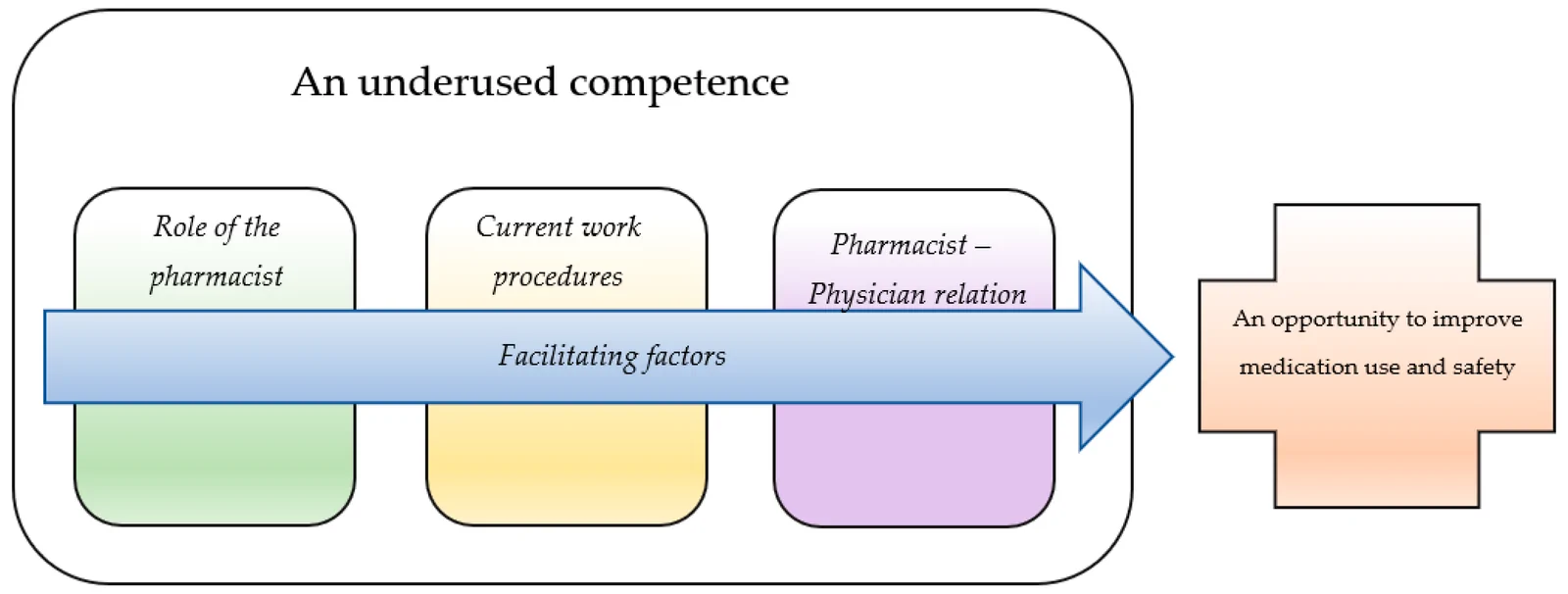
An overall concept integrates the four categories. The three categories of *role of the pharmacist, current work procedures*, and *pharmacist–physician relationship* include barriers experienced by pharmacists when writing referrals. By implementing *facilitating factors* to diminish barriers, an opportunity to improve medication use and safety can be achieved.

Overall, participants viewed referrals positively and considered them a means of improving patient safety during transitions of care. The findings suggest that pharmacists believe they can contribute to patient care with their services during transitions of care. The pharmaceutical service regarding patient care is evolving in Sweden, and this study illuminates issues regarding several barriers that need to be taken into consideration before the referral practice can be fully implemented. The main findings from this qualitative study fit into four categories, whereof three mainly cover identified barriers: *pharmacist role, current work procedures* and *pharmacist–physician relationship*. These barriers can be minimised by implementing the fourth category, *facilitating factors*. In turn, an opportunity to improve medication use and safety can be achieved. This opportunity may remain unrealised if pharmacists’ competence is not fully utilised. An overall concept of an *underused competence* permeates all four categories (Figure 1).

Overall, although broad experience was lacking, the pharmacists perceived the writing of referrals as a positive yet indistinct new task and agreed upon the importance of contributing to patient safety in the transition of care. By participating throughout the hospital stay and becoming fully part of the team, referrals can be incorporated into daily tasks. Feedback from GPs and prioritisation from management were other important stimulating factors.

The respondents described that the role of the pharmacist was perceived as unclear by other professions. Pharmacists’ responsibilities are regulated by the tasks they have, which also reflect the heterogenous role, a finding consistent with previous studies [7,25,26]. By becoming fully integrated in the healthcare team and ongoing patient care, the role and responsibility can be clarified. The fact that pharmacists are not a full part of the team has previously been studied in several countries [7,26,27,28,29]. However, in some countries, such as the UK, Australia and Canada, pharmacists’ responsibilities within healthcare systems have expanded and become more frequent, leading to greater involvement in patient care [2,4,17]. A study from the US found that transition of care pharmacists still identified MRP after discharge despite robust medication reconciliations at discharge, indicating a need for further follow-up post discharge [14]. In the UK, pharmacists’ scope of practice has expanded according to their competence and qualifications. For example, some pharmacists are authorised to prescribe independently within their competence (pharmacist independent prescribing) [30]; in addition, some community pharmacies receive referrals at discharge according to the Discharge Medicines Service programme. All these efforts aim to increase patient safety and reduce the workload of GPs [2,3]. These countries appear to make greater use of pharmacists’ professional competences compared to Sweden. A Cochrane review regarding pharmacist-provided non-dispensing services from middle-income countries reported better patient clinical outcomes (for example, blood pressure, blood glucose), and reduced rates of hospitalisation and visits to general practice [31]. A more recent Cochrane review reported that medication reviews are likely to reduce hospitalisation rates [32].

As pharmacist services in hospitals evolve [33], healthcare organisations and management need to prioritise and be clear about the pharmacist’s role in order to facilitate collaboration for the greatest possible benefit. As for writing referrals, pharmacists’ responsibilities could increase and be more distinct.

The pharmacists placed considerable importance on patient-centred care, and were mindful of the risk that their interventions could undermine patients’ trust in their GP or negatively affect treatment adherence. The fact that patients can read their electronic medical records was also mentioned as a factor to consider on how to express yourself when writing in the medical record. However, patient-centred care should not be a barrier. In the US, for example, pharmacists have taken on greater responsibility in the discharge process by collaborating with patients to determine medication regimens that consider economic constraints, while also providing education to enhance adherence and medication knowledge [14]. By involving the patient in the medication treatment at an early stage, medication use could be adapted in a better way and hopefully improve adherence [34,35]. Pharmacists could play an important role in the discharge process with referrals naturally integrated into this procedure, and support patient care without undermining the GP.

The respondents agreed upon the importance of contributing to patient safety in care transitions, which is also emphasised in several studies from European countries where pharmacist services are well-established [11,36,37,38,39,40]. Pharmacists being thorough and detailed was seen as both an advantage and a disadvantage. Thorough and informative work could facilitate and support physicians’ decision-making. However, it is also time-consuming, and the pharmacists’ effort may not always produce proportionate outcomes for inpatients. The importance of referral for unresolved MRPs should therefore be reinforced to avoid missing potentially clinically relevant recommendations that could prevent patient safety incidents. Previous studies from Sweden and Norway have not demonstrated reduced readmission rates following pharmacist involvement at discharge [6,41], but GPs have been shown to value and act upon recommendations from hospital care [42]. According to the World Health Organisation (WHO), collaboration is necessary to improve care transitions [13]. Several studies emphasise that discharge needs to be prioritised, and that follow-up is especially necessary as many medication adjustments are made during hospital care and close to post-discharge [12,18,43].

In our study, some pharmacists used alternative strategies for information transfer, as referral was a relatively new possibility that had not yet been fully incorporated. This indicates that the need for information transfer exists, but that the model might partly be difficult to implement due to limited pharmaceutical capacity and lack of prioritisation from management. Pharmacist referrals cannot alone prevent readmissions but may be one useful tool in the discharge process to improve medication use and safety. The fact that there are few pharmacists causes limited capacity and poor continuity, which in turn leads to underutilised pharmacist services. Medication reconciliation is prioritised over other tasks by the management since medication discrepancies at admission are common. These factors inhibit referrals and discharge needs to be prioritised. In the future, management could benefit by taking pharmacists into account when optimising the discharge process.

The pharmacists perceived that physicians from different departments acted to different degrees on identified MRPs. Physicians in surgical departments tended to primarily address acute MRPs, which should imply a greater need to mediate MRPs to GPs. Indeed, a higher proportion of medication discrepancies in discharge summaries from surgical departments have previously been shown [44]. Although these facts were mentioned by several pharmacists, it did not seem to influence the number of written referrals from surgical departments. This was explained by limited capacity and continuity. Some physicians who were informed about referrals encouraged pharmacists to send referrals, rather than carry out some of the recommendations during hospital admission. This approach may enable a more effective utilisation of pharmacists’ competence by allowing pharmacists to take greater responsibility for MRPs. While there may be a risk of excessive workload for GPs described by researchers in the UK [45], a previous Swedish qualitative study reported that GPs were receptive to information provided by pharmacists [36]. Our study indicates that pharmacists need to take a greater responsibility and assess where an MRP should be addressed. By becoming integrated into the daily healthcare team and participating in the discharge process, referrals could be facilitated. The pharmacist would be able to take more responsibility and relieve the physician, which is also confirmed by others [25]. This approach may facilitate the utilisation of a currently underutilised pharmacist competence. Increasing the number of pharmacists in primary care who work collaboratively within healthcare centres could also support GPs and enhance the quality of MRP follow-up after discharge.

In our study, a varied view of the recipient (GPs) emerged. Many pharmacists felt uncertain about their attitude, but at the same time there was a perception that GPs welcomed pharmacist information. Although previously demonstrated in several studies, it needs to be conveyed to pharmacists [36,39,46,47,48]. In our study, the importance of feedback from GPs to stimulate referral was emphasised. But for this to be done, the GPs must be able to mediate feedback in a simple way. Simultaneously, an Australian study by Foot et al. described uncertainty from GPs regarding the role of pharmacists; a fear also expressed by the pharmacists in our study [48]. However, since referral is a request for a specific medical service, this might clarify the pharmacist’s task to the GP to some extent. Some pharmacists in our study mentioned that if referrals could be sent to pharmacists in primary care, the feeling of uncertainty would diminish. Studies from the UK, US, Australia and Canada found that pharmacists who work with transition of care and perform a follow-up or a medication review after discharge still identify new MRPs, which might highlight the need for pharmacist–pharmacist referrals [12,14,18,49]. This is, however, not yet possible in our setting since pharmacists are not standard healthcare personnel in primary care. Future studies in our setting would be of interest, evaluating post-discharge follow-up by pharmacists in primary care, to potentially improve medication safety in transition of care and to fully utilise pharmacists’ competencies.

If the expertise of clinical pharmacists and their capacity to ensure the transfer of adequate medication-related information across care transitions are not fully utilised, important opportunities to optimise medication use and enhance patient safety may be lost. It is therefore essential that healthcare organisations and leaders facilitate the effective integration of pharmacists into multidisciplinary care teams. In addition, newly established referral and information-transfer procedures need to be recognised and supported throughout the organisation. This is particularly important for pharmacists, a professional group that has not traditionally issued referrals. Ensuring that these procedures are widely understood and embedded in clinical practice may promote the successful implementation. Failure to do so risks leaving considerable potential unrealised, both in terms of economic efficiency and improvements in patient safety.

### 4.1. Strengths and Limitations

To our knowledge, clinical pharmacists’ experiences of the Swedish pharmacist-led referral model, which operates alongside traditional discharge processes, have not previously been explored in depth. By using focus group discussions and individual interviews with criterion-based participant inclusion, our study captured perspectives from participants who were considered representative of the study population, which strengthens the credibility of the findings. However, although the participants were representative of our county, the findings may not be transferable to other settings and countries with different healthcare systems and conditions. The results contribute to illuminating the importance of the barriers and facilitating factors perceived by pharmacists. As noted above, similar findings have been reported in comparable settings, which may support the transferability of the results to similar contexts [25].

Another limitation in this study was the accidental partial loss of the recording from Focus Group 2, which may have influenced the collected data. However, a summary of the discussion was sent to the participants for verification, and they were encouraged to provide feedback on whether any additions or amendments were necessary and whether the summary accurately reflected the discussion. No further additions or corrections were needed.

As for reflexivity, the research team had a composition of pharmacists (KW, CL) with a pre-understanding of clinical pharmaceutical care in hospitals (such as identifying MRP) and two GPs (GC, SM) with experience of referrals and collaboration with pharmacists. KW, who shared the same profession as the participants and was known to them through a regional network of clinical pharmacists, may have contributed to affinity bias and social desirability bias (by respondents wanting to provide responses in favour of the interviewer). However, the richness and depth of the data, including both barriers and facilitators, is considered a minor limitation. The presence of CG as a co-moderator (profession as a GP), not known by the participants, may have mitigated these potential biases as well as the risk of implicit assumptions influencing data collection and interpretation. Although the multidisciplinary composition of the research team was a strength throughout both data collection and analysis, our pre-understanding may also have influenced the interpretation of the findings, a challenge that has been described by Korstjens et al. [50].

### 4.2. Future Research

Researchers from various countries have studied pharmacist services within their contexts with activities such as pharmacists as authorised prescribers to reduce workload for GPs as well as taking an active role in the transition of care [2,3,7]. Given that healthcare systems are organised differently across countries, it is important to study interventions within local contexts. For example, UK pharmacists have the possibility to have extended responsibilities [30], while in the US medication financing is determined by a mix of private insurance, public programmes and intermediaries [51]. Further research is needed to study the clinical significance of the content in pharmacist referrals and whether GPs in primary care act on the communicated recommendations. Understanding GPs’ perceptions of the service provided by pharmacists using, for example, qualitative research or surveys, would also contribute to the implementation process. Over the long term, patient outcomes such as hospital readmission could be of interest to assess and emphasise the importance of whether the referral process should be integrated in hospital practice.

## 5. Conclusions

Pharmacists perceived that they can contribute to patient safety during care transitions. However, the use of pharmacist referrals for identified MRPs was hindered by limited integration into healthcare teams, insufficient continuity, and the novelty of the referral procedure, which often involved unfamiliar collaboration partners. Fundamental organisational rearrangements, such as increased pharmacist continuity and clearer role definitions, are needed to facilitate implementation of the referral process. Further research is needed to understand the impact of referrals on medication treatment and patient outcomes.

## Figures and Tables

**Table 1:** Description of focus group interviews and individual interviews, and participating pharmacists.

Characteristics	Focus Group 1	Focus Group 2	Focus Group 3	Focus Group 4	Individual Interviews 1–3
Interview duration, minutes	49	81	92	79	43, 47, 58 *
Male/Female	0/3	0/5	2/2	0/4	0/3
Experience from writing referrals	1	4	1	3	2
Time in profession					
<1 year	-	1	-	-	-
≥1<5 years	-	1	1	2	1
≥5<10 years	-	2	1	1	-
≥10 years	3	1	2	1	2

* Interview durations are reported from the shortest to the longest and do not reflect the chronological order in which the interviews were conducted.

**Table 2:** Scheme over and example of meaning units condensed into codes.

Meaning Unit	Condensed	Code	Subcategory	Category
It is probably easier to get started when you feel that you are part of the team. This also facilitates clearer communication with the physician and nursing groups, enabling better alignment and coordination. As a result, collaboration improves, and certain issues can be managed independently.	Being part of the team at the clinic can facilitate to start writing referrals	Teamwork can facilitate writing referrals	Being part of the team	Facilitating factors
This is not a role that absolutely has to exist at the ward; we [pharmacists] are more like extra support that exists in certain places; it is not mandatory to have a pharmacist.	No responsibilities since pharmacists are not mandatory in the clinic	Pharmacists not mandatory in the clinic results in unclear role	Unclear pharmacist role and responsibilities	Role of the pharmacist

**Table 3:** Scheme of the subcategories grouped into categories.

Category	Subcategory
*Role of the pharmacist*	Unclear pharmacist role and responsibilities New and unaccustomed work procedure New role in relation to the patient Pharmacists’ thoroughness for better and worse No wish to be a control function
*Current work procedures*	Management and prioritisation influence Not involved in discharge procedure Lack of continuity Alternative strategies Suitable MRPs for referral
*Pharmacist—physician relationship*	Physician and medical specialty influence Physicians’ actions to recommendations Pharmacist referrals versus physician referrals Uncertainty in relation to receiver
*Facilitating factors*	Instructions and introductions Being part of the team Communication and feedback

## Data Availability

The data presented in this study are available on request from the corresponding author due to large text files based on focus group discussions and individual interviews.

## Supplementary Materials

The following supporting information can be downloaded at: https://www.mdpi.com/article/10.3390/pharmacy14050109/s1. File S1: COREQ (COnsolidated criteria for REporting Qualitative research) Checklist; File S2: Interview guide.

## Abbreviations

MRP: Medication-related Problem; GP: General Practitioner; FG: Focus Group; WHO: World Health Organisation; UK: United Kingdom; US: United States.
