# Supplementary material for: An Underused Competence: Clinical Pharmacists’ Experiences of Writing Referrals in Transitions of Care
Source: Pharmacy (Basel). 2026 Jul 23;14(5):109. doi: 10.3390/pharmacy14050109 (PMC13516502; doi:10.3390/pharmacy14050109)
Supplement: Supplementary file 1 [file pharmacy-14-00109-s001.zip › File S2. Interview guide.pdf]

Interview guide

Presentation round the table and answer if you have written a referral

Experiences and perceptions

- Tell us about your experiences from referrals regarding DRPs from you as a clinical pharmacist to the GP

- Experiences?
- Others?
- Other strategies?

- Discuss if any benefits or barriers writing the referral yourself? In that case which?

- Role in the team? At the clinic? Continuity and responsibility
- Role towards the patient? New?
- Role towards the hospital physician?
- When is referral adequate and not?
- Shift of/strengthen the role?

Facilitating factors and barriers of the referral process

- Why do you think (so) few referrals are written?
- Discuss which factors facilitate writing referrals?

- Work procedure/workflow?
- New procedure – influence on how often referral is written?
- Various specialties/departments – any difference to write referral?
- Importance of management/managers for pharmacists' work with information transfer?
- What support is needed to write referral?
- What future efforts do you wish for to facilitate the referral procedure?
- Communication routes at the clinic (allocated time?)
- Perceptions of the recipient (GP)? Feedback?
- Unfamiliarity/ Fears?

- What role (do you think) does clinical pharmacists have to increase patient safety regarding information transfer in transition of care?

Summary

- What is the most important to highlight regarding referrals from clinical pharmacists?
- Summary
- Did we miss anything? Is there anything that hasn´t been covered?
